# High-resolution imaging of diabetic retinopathy lesions using an adaptive optics retinal camera

**Published:** 2019

**Authors:** Irina-Elena Cristescu, Radu Ochinciuc, Florian Balta, Leon Zagrean

**Affiliations:** * Retina Clinic, Bucharest, Romania; ** Department of Physiology, Faculty of Medicine, “Carol Davila” University of Medicine and Pharmacy, Bucharest, Romania; *** Clinical Ophthalmology Emergency Hospital, Bucharest, Romania; **** Department of Ophthalmology, Faculty of Medicine, “Victor Babes” University of Medicine and Pharmacy, Timisoara, Romania; ***** Department of Ophthalmology, Faculty of Medicine, “Victor Babes” University of Medicine and Pharmacy, Timisoara, Romania

**Keywords:** adaptive optics retinal camera, diabetic retinopathy, microaneurysms, hemorrhages, hard exudates

## Abstract

**Purpose.** Adaptive optics (AO) imaging is a promising high-resolution investigation technique in ophthalmology that can bring new information about the pathophysiology of diabetic retinopathy.

**Material and methods.** Seven patients previously diagnosed with diabetic retinopathy were investigated with optical coherence tomography (OCT) scanning, OCT angiography, fundus photo, and AO retinal camera (rtx1TM, Imagine Eyes, Orsay, France).

**Results.** The red lesions on fundus photos appeared on AO imaging as hyporeflective lesions. OCT angiography helped us to differentiate between microaneurysms and hemorrhages. Hard exudates had a heterogeneous granular appearance. Retinal oedema was proved to have a blurring effect on the AO images. In addition to this, cystic spaces were identified to have a hyporeflective demarcation line.

**Conclusions.** AO imaging is offering a fine documentation of retinal lesions and might become an important instrument for early diagnosis of diabetic retinopathy and for explaining its pathophysiological mechanisms.

**Abbreviations:** AO = adaptive optics, AOO = adaptive optics ophthalmoscopy, SS = swept source, OCT =optical coherence tomography, SLO = scanning laser ophthalmoscope

## Introduction

Retinal damage from diabetes mellitus is a frequent sight threatening condition globally [**1**,**2**]. The type and duration of diabetes, blood pressure and glycemia are strongly linked to the onset and progression of diabetic retinopathy. Both microvascular [**3**] and neurodegenerative components notably contribute to vision loss [**4**]. Microvascular lesions encountered in non-proliferative diabetic retinopathy consist of thickening of the basement membranes, pericyte loss, capillary occlusion, microaneurysms [**5**]. They lead to retinal ischemia, macular oedema, and retinal neovascularization. The grading of diabetic retinopathy depends on the semi-quantitative assessment of topographical and morphological retinal lesions. In the medical retina units, these are usually assessed through direct eye exam or teleophthalmology by fundus photo or optical coherence tomography scanning [**6**]. Nevertheless, besides the high resolution and contrast of the optical coherence tomography, the detection of small size and low contrast retinal lesions might be a challenge due to the optical aberrations occurring from the anterior segment. The early identification of such diabetic lesions could be valuable in the early diagnosis of diabetic retinopathy. Moreover, studying the turnover of these lesions by a noninvasive procedure might be a relevant parameter of vascular disease processes. 

Since the 2000s, a new imaging tool was able to deal with these challenges. Adaptive optics (AO) enhanced ophthalmoscopy (AOO) can compensate for the optical faults in vivo with a wavefront sensor, a deformable mirror, and a control system. AOO has been used with retinal cameras, optical coherence tomography (OCT) and scanning laser ophthalmoscopes (SLO) [**7**].

With AO retinal camera photoreceptors and retinal vascular changes in diabetes [**8**,**9**] have been assessed. Nonetheless, the general knowledge on the appearance of the diabetic retinopathy lesions on AO camera and other high resolution imaging tools needs to be enhanced. 

In this study, we presented qualitative changes of diabetic retinopathy using the rtx1 AO camera (Imagine Eyes, Orsay, France), swept source (SS) OCT, OCT angiography, and fundus photo. 

## Materials and Methods

The study was conducted in accordance to the Declaration of Helsinki and the patients gave their informed consent to participate according to the Romanian legislation. All investigations were part of the routine assessment of diabetic retinopathy in the clinic. 

Study Participants. Seven patients with diabetes mellitus (five males and two females) and diabetic retinopathy lesions who received ophthalmological services at the Retina Eye Clinic were included in the study. The inclusion criteria the subjects had to meet in order to enter the study were: minimum 18 years old, diagnosis of type I or II diabetes mellitus as defined by the American Diabetes Association [**10**], stable central fixation and clear optical media. Subjects with retinal pathology of non-diabetic aetiology were excluded. 

Examination. All the patients underwent a routine ophthalmological evaluation including the measurement of best corrected visual acuity on ETDRS charts, slit lamp examination of the anterior and posterior segment of the eye and intraocular pressure measurement. Dilation of the pupil was pharmacologically induced by Phenylephrine 10% and Tropicamide 1%. This was followed by retinal imaging including SS OCT (DRI OCT Triton, Topcon), OCT angiography (SS OCT Angio, Topcon), standard field color fundus and red free photography (DRI OCT Triton, Topcon). Axial length was measured using optical biometry (Aladdin, Topcon). Diabetic retinopathy lesions were identified by the investigators based on fundus photos, slit lamp examination, OCT and OCT angiography scans.

Further on, we acquired a series of images of the retina using AO retinal camera (rtx1TM, Imagine Eyes, Orsay, France). One final image with an improved signal to noise ratio was the result of 40 processed raw images of the same retinal area using the software provided by the manufacturer. The patients were instructed to fixate a cross in the eyepiece of the camera. Its coordinates were set according to the spatial distribution of the lesions of interest, so that those were included in the 4x4 degrees field of view. The cone mosaic was obtained with the software provided by the manufacturer, i2k Retina AO, Imagine Eyes, France, which enables stitching multiple images obtained using rtx1 AO retinal camera.

Image analysis. All image interpretations were achieved by the same investigator, trained in advance on retinal image analysis.

## Results

Microaneurysms and dot hemorrhages were the most common lesions met in the studied retinas. Hard exudates were accompanied by retinal oedema on the SS-OCT. Nevertheless, we could not identify any cotton wool spots in any patient.

**Fig. 1:**
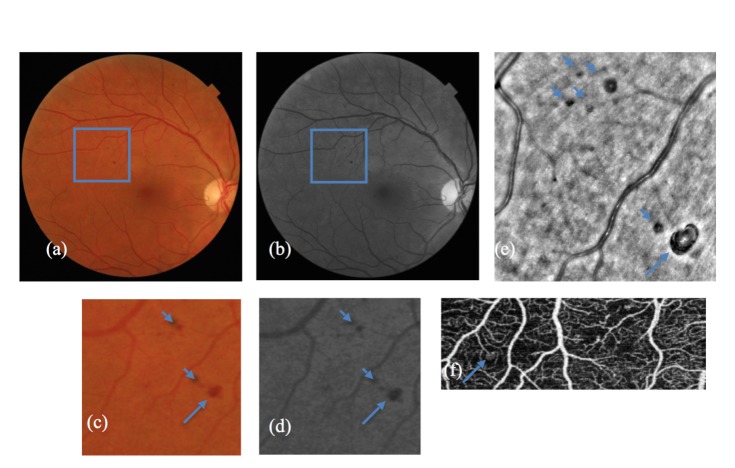
(a), (b) Color fundus photo and red free photo of a patient with microaneurysms and haemorrhages. (c), (d) Larger magnification of the area delimited in the previous photos, (a) and (b). The big arrow indicates a microaneurysm and the arrowheads show haemorrhages. (e) Adaptive optics image corresponding to (c) and (d). The black lesion with inner hyperreflectivity marked by a big arrow is a microaneurysm. The black lesions marked by small arrows are retinal haemorrhages. (f) OCT angiography revealed only one lesion from the ones above which is the microaneurysm

The microaneurysms presence on AO images was confirmed by OCT angiography scans, fundus photos and by SS-OCT scans (as blocking factors). Hemorrhages were identified as hyporeflective lesions with distinct margins. Although hemorrhages could not be distinguished from microaneurysms in color fundus photos and red free image, they could be discriminated by OCT angiography and by the lack of hyperreflective areas on AO images (**Fig. 1**). 

Hard exudates on AO images had a heterogeneous appearance with distinct dark margins and with deposit clumps of different sizes. Hyperreflective areas might be due to lipid deposits, whereas the hyporeflective ones might be the shadows of the first ones on the photoreceptors.

**Fig. 2:**
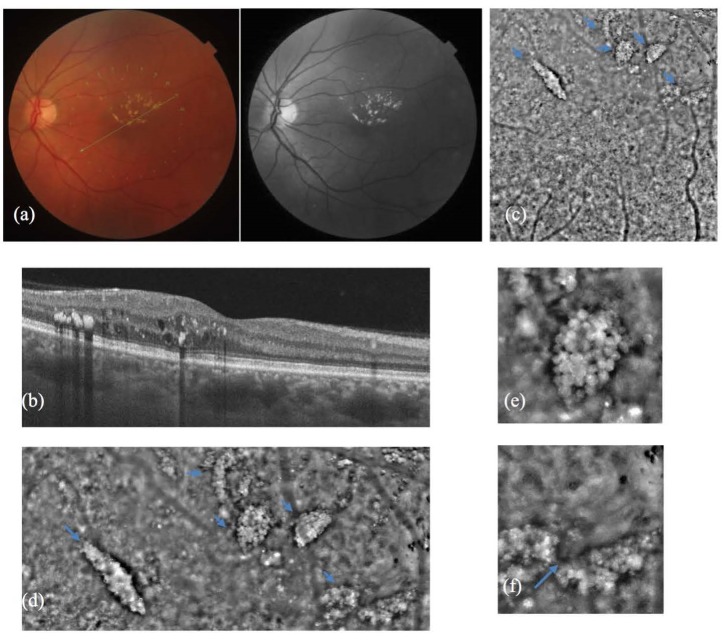
(a) Color fundus photo and red free photo of a patient with hard exudates and retinal oedema. (b) Optical coherence tomography (OCT) corresponding to the green line in (a) shows hard exudates in the middle retinal layers. (c) Adaptive optics imaging cone mosaic; small arrows indicate the hard exudates. (d) magnification of the upper part of (c) in which besides the hard exudates (small arrows), oedema blurring the retinal image can be noticed. (e) magnification of (d), detail of a hard exudate showing foci of hyper and hyporeflectivity. (f) magnification of (d), detail of two hard exudates and retinal oedema; the cystic spaces have a sharp demarcation line indicated by the big arrow

Retinal oedema proved to blur the AO images. Also, cystic spaces were identified to have sharp demarcation lines (**Fig. 2**).

## Discussion

This present study aimed to document the early lesions of diabetic retinopathy by AO retinal camera. It comes as a completion to previous qualitative studies of diabetic retinopathy lesions as observed by AO rtx1 retinal camera [**11**]. Thus, in this study we included angio OCT as an investigation tool, valuable in identifying microaneurysms noninvasively. 

For each type of lesion, we had minimum six identical observations, the conditions of a significant binomial experiment for qualitative features being met. 

Adaptive optics is a promising tool for the detection of microvascular lesions. In experimental studies, histology proved that pericytes and endothelial cells loss of the retinal vessels occur before any clinically detectable lesions of diabetic retinopathy [**12**]. Being the first clinically detectable signs of diabetic retinopathy, microaneurysms play an important role in the early diagnosis of diabetic retinopathy. Early changes of perifoveal capillaries have been documented by AOO [**13**-**15**]. Moreover, a morphological grading of microaneurysms in six categories was obtained with AO scanning light ophthalmoscope fluorescein angiography [**15**]. Nevertheless, the presence but not the morphology or size alone of microaneurysms was proved to be a sensitive instrument for the follow-up of the retinal pathology. Besides describing the walls of microaneurysms, the same team found hypofluorescent infiltrations in one third of the studied microaneurysms that, according to histological studies, could be inflammatory cells, erythrocytes breakdown products, or cellular waste. More recently, these lesions have been studied with tridimensional imaging modalities using adaptive optics optical coherence tomography (AOOCT) [**16**]. This tool made it possible to establish that microaneurysms located in the inner nuclear layer had connections to the intermediate or deep capillary plexus. Moreover, on AOOCT images, fibrosis or cell infiltration corresponds to the hyperreflective material found in more than half of the studied microaneurysms [**17**]. Consequently, hyperreflective foci of the microaneurysms in this study were congruent to previous findings. 

However, a sophisticated documentation of lesions from more advanced stages of diabetic retinopathy is also accomplished with AO imaging. 

As Bek documented [**11**], some dark hyporeflective homogenous elements confirmed as hemorrhages could be resolved by AO retinal camera, but not by fundus imaging. This suggests that this investigation tool might be useful in the early detection of diabetic retinopathy changes. 

Hard exudates were visible in SS-OCT, red free images and color fundus photography. In AO imaging, hard exudates appeared as dark bordered heterogeneous grainy lesions with hyper and hyporeflective areas (high reflectance from lipids deposits). The clumps of various sizes might correspond to the places where plasma proteins and/ or lipoproteins are deposited or reabsorbed. In an OCT study [**18**], it has been concluded that the hyperreflective foci are precursors of hard exudates that accumulate in the outer plexiform layer. High resolution imaging by AOSLO made possible defining two morphologically different categories of hard exudates [**19**]. A more detailed study of the morphology and changes in time of hard exudates or after antiVEGF therapy might increase the understanding of the pathophysiology of macular oedema in diabetes. 

To sum up, the capacity to document retinal lesions of diabetic retinopathy *in vivo* at histological resolution using AO imaging has been offering a high level of documentation. Thus, this new method might help clinicians detect early retinal changes and effects of different therapeutic methods that are not visible with classical techniques in diabetic patients and better explain the mechanism of the disease. Future observational studies of the dynamic changes of the diabetic lesions could be an important step in order to create an examination protocol for diabetic patients that includes AO imaging. 
